# Potential Probiotic *Enterococcus faecium* OV3-6 and Its Bioactive Peptide as Alternative Bio-Preservation

**DOI:** 10.3390/foods10102264

**Published:** 2021-09-24

**Authors:** Thiwanya Choeisoongnern, Sasithorn Sirilun, Rungaroon Waditee-Sirisattha, Komsak Pintha, Sartjin Peerajan, Chaiyavat Chaiyasut

**Affiliations:** 1 Department of Pharmaceutical Sciences, Faculty of Pharmacy, Chiang Mai University, Chiang Mai 50200, Thailand; thiwanya_c@cmu.ac.th; 2 Innovation Center for Holistic Health, Nutraceuticals, and Cosmeceuticals, Faculty of Pharmacy, Chiang Mai University, Chiang Mai 50200, Thailand; 3 Department of Microbiology, Faculty of Science, Chulalongkorn University, Bangkok 10330, Thailand; rungaroon.w@chula.ac.th; 4 Division of Biochemistry, School of Medical Sciences, University of Phayao, Phayao 56000, Thailand; komsakjo@gmail.com; 5 Health Innovation Institute, Chiang Mai 50200, Thailand; s.peerajan@gmail.com

**Keywords:** probiotics, Enterococci, cell adhesion, immunomodulatory, antimicrobial peptides, bacteriocin, ribosomal proteins, bio-preservation

## Abstract

Probiotic *Enterococcus faecium* OV3-6 and its secreted active peptide were characterized and investigated. The strain survived in simulated gastric and small intestinal conditions at 88.16% and 94.33%, respectively. The safety assessment revealed that the strain was shown α-hemolysis and susceptible to most clinically relevant antibiotics, but intermediate sensitivity to erythromycin and kanamycin was found. It does not harbor any virulence genes except for the *efaA_fm_* gene. Both of its living cells and the cell-free supernatants (CFS) of the strain significantly reduced the adhesion of *E. coli* and *S. Typhi* on Caco-2 cells. The strain can regulate the secretion of pro and inflammatory cytokines, IL-6 and IL-12 and induce the secretion of anti-inflammatory IL-10 of the Caco-2 cell. The strain can prevent the growth of Gram-positive strains belonging to the genera *Bacillus*, *Carnobacterium*, *Listeria*, and *Staphylococcus*. It also presented the *entP* gene that involves the production of bacteriocin named enterocin P. The antimicrobial peptide was matched 40% with 50S ribosomal proteins L29 (7.325 kDa), as revealed by LC-MS/MS. This active peptide exhibits heat stability, is stable over a wide pH range of 2−10, and maintains its activity at −20 and 4 °C for 12 weeks of storage. Altogether, *E. faecium* OV3-6 thus has potential for consideration as a probiotic and bio-preservative for applied use as a fermented food starter culture and in functional food or feed industries.

## 1. Introduction

The contamination of undesirable microorganisms in foods is the initial problem that leads to serious foodborne illness cases in humans and the loss of product in food industrials. Particularly, local fermented foods have been highly contaminated by undesirable microorganisms and toxins [1]. The government regulations and consumer demands push the food manufacturers to find the natural preservatives to control the contaminating microbes primarily responsible for food spoilage and poisoning.

Lactic acid bacteria (LAB) are Gram-positive cocci or bacilli, aerotolerant, homofermentative or heterofermentative, and lactic acid producers [2]. LAB has been used in starter culture that plays a vital role in fermentation as flavorings and texturizing or preservative agents [3]. Interestingly, the metabolites of LAB exert antimicrobial activity. Because of generally regarded as safe (GRAS) status, the use of LAB or their metabolites as a natural preservative in food has gained much importance [3]. Several LABs have played a role as potential probiotics as live microorganisms, which, when administered in adequate amounts, confer a health benefit on the host [4]. Most human probiotic belongs to of *Lactobacillus* spp. and *Bifidobacterium* spp. [5], whereas less information exists about the effectiveness of enterococcal strains as probiotics. Enterococci belong to the LAB group, which is widespread in foods and the environment, and they inhabit the gastrointestinal (GI) tract of mammals and other animals [6]. It has been recognized as the leading cause of hospital-acquired infections [7]. To date, the genus *Enterococcus* has not yet obtained the status GRAS. However, *Enterococcus* strains are currently recognized as probiotic and commercially available [8]. Potential probiotics have been reported to modulate gut microbiota, inhibit pathogens adhesion, increase gastrointestinal barrier function, induce anti-inflammatory cytokine and suppress pro-inflammatory cytokines [9], and to have cholesterol-lowering activity [10]. However, enterococci are associated with vancomycin resistance and nosocomial infections. Therefore, to ensure the safety and use of enterococci in foods or as probiotics there must be enough data based on case-by-case studies.

Enterococci usually produce many substances with antimicrobial activity, including antimicrobial peptides (AMPs) known as bacteriocins. Bacteriocins are small, ribosomal synthesized, extracellularly released bacterial peptide or protein molecules that can kill some closely-related bacteria [11]. However, nisin from *Lactococcus lactis* is one commercially produced bacteriocin widely used as a food additive [12]. These AMPs represent an alternative to antibiotic treatment and can also inhibit foodborne pathogens by forming pores in the membrane of target cells, which are the main mechanisms of its action [13].

Besides bacteriocin, ribosomal proteins (RPs) also have an antimicrobial activity [14]. RPs are components of the small and large subunit of ribosomes, which act like the machinery for protein synthesis in prokaryotic and eukaryotic cells. L27 and L30 proteins are a component of the 50S ribosomal subunit secreted from the *Lactobacillus salivarius* SGL 03 strain that can function as AMPs [15]. However, the survey of antimicrobial activity of RPs remains incomplete, and information as such is extremely limited [14].

The present study aimed to investigate and characterize the antimicrobial activity, adhesion properties, immunomodulatory and secreted AMPs, and potential active peptide secreted from probiotic *E. faecium* OV3-6 for its future intended use in the food/feed industry.

## 2. Materials and Methods

### 2.1. Microbial Strains Cultivation

The probiotic strain of *E. faecium* OV3-6 (GenBank accession number: MN453594) was isolated from fermented plant (*Houttuynia cordata* Thunb.) beverages, and the key probiotic properties were evaluated and collected in Chiang Mai, Thailand [16]. The strain was propagated in de Man Rogosa and Sharpe (MRS) medium at 37 °C. Antimicrobial tested strains included *Listeria ivanovii* SLCC 2379 were obtained from Institute National de la Recherche Agronomique (INRA), Nantes, France. *Brochothrix thermosphacta* DSMZ 20171^T^, *Carnobacterium maltaromaticum* NCDO 2760, *Staphylococcus aureus* CIP 76.25, *Escherichia coli* CIP 76.24, and *Listeria innocua* CIP 80.11^T^ were obtained from Ecole Nationale Nantes Atlantique Vétérinaire, Agroalimentaire et de l’Alimentation (ONIRIS), Nantes, France. *Bacillus cereus* ATCC 11778, *Clostridium perfringens*, *Cronobacter sakazakii* ATCC BAA-894, *Escherichia coli* ATCC 5922, *Propionibacterium acnes* ATCC 6919, *Pseudomonas aeruginosa* ATCC 27853, *Salmonella* Typhi DMST 22842, *Salmonella* Typhimurium TISTR 1469, *Shigella sonnei*, *S. aureus* ATCC 5923, *Staphylococcus epidermidis* ATCC 12228, *Streptococcus mutans* ATCC 25175, *Vibrio harveyi* ATCC BAA-1119 and *Candida albicans* ATCC 90028 were obtained from the culture collection of Faculty of Pharmacy, Chiang Mai University (CMU), Thailand. The tested strains were cultured in Brain Heart Infusion (BHI) medium at 37 °C, except *B. thermosphacta* was grown at 25 °C, and yeast was cultured in Sabouraud Dextrose Broth (SDB). In PCR amplification of virulence and bacteriocin genes, *Enterococcus* spp. from CMU, *Enterococcus faecalis* CM6CR07 and *E. faecalis* CF1GI14, which kindly provided by Hwanhlem [17], were grown in MRS medium and used as a positive control. All microorganism culture mediums used in this study were punched from Himedia (Mumbai, India).

### 2.2. Probiotic Properties and Safety Assessment

#### 2.2.1. Survival in Simulated Gastric Juice and Small Intestinal Condition

The culture of *E. faecium* OV3-6 (18 h) was harvested by centrifugation (4 °C at 10,000× *g* for 5 min). The cell pellet was resuspended in simulated gastric juice containing 3 g/L pepsin (Sigma-Aldrich, St. Louis, MO, USA) in phosphate-buffered saline (PBS) pH 2.5 and incubated at 37 °C for 0, 1, 2, 3, and 4 h. Aliquots of 0.1 mL were taken and then bacteria were washed with PBS pH 7.0 to determine the total viable count on MRS agar. After incubating at 37 °C for 24 h, the survival cell was enumerated. The percentage survival of the bacteria was calculated as Equation (1) [18], where N1 is the number of initially inoculated viable cells (CFU/mL), and N2 is the number of survived viable cells (CFU/mL).

% survival = (logN2/logN1) × 100(1)

For the small intestinal condition tolerance, the cell pellet was resuspended in PBS solution containing 0.3% (*w*/*v*) bile salts (Sigma-Aldrich) and 1 mg/mL pancreatin (Sigma-Aldrich), pH 8.0. The bile salt resistant cell was assessed after incubation at 37 °C for 0, 1, 2, 4, and 6 h. Aliquots of 0.1 mL were taken and then bacteria were washed and neutralized with PBS pH 7.0. The total viable cells was enumerated on MRS agar after incubation at 37 °C for 24 h. The percentage survival of the bacteria was calculated as Equation (1) [18].

#### 2.2.2. Bile Salt Hydrolase (BSH) Activity

The bacteria culture (16 h) was streaked on MRS agar, which was supplemented with 0.5% (*w*/*v*) sodium salt of taurodeoxycholic acid (TDCA) (Sigma-Aldrich) and 0.37 g/L of calcium chloride (UNILAB, Sydney, NSW, Australia). Plates were incubated at 37 °C for 72 h. The presence of precipitated bile acid surrounding colonies (halo zone) indicated the positive bile salt hydrolase activity of bacteria [19].

#### 2.2.3. Hemolytic Activity

The bacteria culture (16 h) of *E. faecium* OV3-6 was streaked on sheep blood agar. The plate was incubated at 37 °C for 48 h. After incubation, the hemolytic reaction was observed in appearance zones around colonies. The absence of zones and greenish zones was interpreted as γ-hemolysis and α-hemolysis (negative hemolytic activity). When presented, the clearing zone around colonies was considered as β-hemolysis (positive hemolytic activity) [20].

#### 2.2.4. Determination of Antibiotic Susceptibility

Antibiotic susceptibility of *E. faecium* OV3-6 was determined by the disk diffusion method [18,21]. Briefly, the test bacteria were grown in a broth culture medium for 18 h before the test, and the bacteria cell was resuspended in PBS (0.5 McFarland equivalent to 10^8^ CFU/mL). Bacteria suspension was swabbed on Mueller-Hinton agar (MHA) (Himedia). The antibiotic disks consisting of 10 µg ampicillin, 30 µg chloramphenicol, 2 µg clindamycin, 15 µg erythromycin, 10 µg gentamicin, 30 µg kanamycin, 10 Unit penicillin G, 25 µg streptomycin, 30 µg tetracycline, and 30 µg vancomycin were placed on the agar plate by aseptic technique. All the antibiotics disks in this study were purchased from Himedia. After incubation at 37 °C for 24 h, the growth inhibition zone diameters (mm) around the disk were measured. The results were interpreted according to the breakpoints recommended by The Clinical and Laboratory Standards Institute (CLSI, 2016) guidelines. With the exception of kanamycin, gentamicin, streptomycin, and clindamycin were interpreted according to breakpoints proposed by Nueno-Palop [21].

#### 2.2.5. Evaluation of Virulence-Associated Genes

The presence of virulence-associated genes was evaluated by polymerase chain reaction (PCR). The genomic DNA of *E. faecium* OV3-6 was extracted by using the NucleoSpin^®^ Microbial DNA kit (Macherey-Nagel, Düren, Germany). The primer and amplification conditions were performed following in Table S1. The target genes were *ace* (adhesin of collagen protein), *asa1* (aggregation substance), *cylA*, *cylB* (cytolysins), *efaA_fs_* (cell wall adhesion in *E. faecalis*), *efaA_fm_* (cell wall adhesion in *E. faecium*), *esp* (enterococcal surface protein), *hyl* (encodes the hyaluronidase), and *gelE* (encodes the protease GelE with gelatinase activity) [11,22,23]. The PCR mixtures were performed in 25µL final volume containing 50 ng of DNA template, 0.25 µM of each primer, 2.5 µL of 10× PCR buffer containing 35 mM MgCl_2_, 1 U of Taq DNA polymerase, 2.5 µL of dNTPs mixture (2.5 mM each). All PCR reactions were purchased from iNtRON (iNtRON Biotechnology, Inc., Gyeonggi, South Korea). Amplifications were carried out by using DW-T960 Smart Gradient PCR Thermal Cycler (Drawell, Shanghai, China) with the condition of initial denature at 94 °C for 5 min, followed by 35 cycles of denaturation at 94 °C for 1 min, annealing at an appropriate temperature for 1 min, extension at 72 °C for 1 min and a final extension step of 5 min at 72 °C. The DNA from strains *E. faecalis* EFA70 and EFA14 were used as a positive control. Amplification products were separated on a 1.5% agarose gel containing RedSafe™ nucleic acid staining solution (iNtRON) in 1× TAE buffer for 35 min at 100 V. And made visible by UV trans-illumination.

### 2.3. Inhibition of Adhesion on Caco-2 Cell Model

#### 2.3.1. Cell Culture

The Caco-2 cell line (No. RCB0988) (RIKEN BioResource Research Center, Ibaraki, Japan) were cultured in Dulbecco’s Modified Eagle’s Medium (DMEM) (Gibco, NY, USA) containing 10% (*v*/*v*) fetal bovine serum (FBS) (Gibco), 2 mM L-glutamine (Gibco), and 1% (*v*/*v*) antibiotic solution (100 U/mL penicillin and 100 µg/mL streptomycin) (Gibco). The cell was incubated at 37 °C under a 5% CO_2_ atmosphere and 95% relative humidity. For bacteria binding to Caco-2 cells and inhibition of adhesion assays, the cell was placed in 24-well plates to obtain semiconfluent monolayers for 72 h [24]. The culture was replaced with antibiotic-free and FBS-free fresh DMEM for 24 h before the assay was performed and used at ≥80% confluence to avoid association with the well [25].

#### 2.3.2. Inhibition of Enteropathogenic Bacterial Adhesion

The binding of enteropathogenic *E. coli*, *S. Typhi*, and *E. faecium* OV3-6 to Caco-2 intestinal epithelial cells was studied using a previously modified method [26]. Briefly, Caco-2 cells were treated with 0.2 mL overnight in culture bacteria (10^7^ CFU/mL) in a cell medium without antibiotic and FBS at 37 °C under a 5% CO_2_ atmosphere and 95% relative humidity for 1 h. The Caco-2 cells were washed with PBS to remove unbound bacteria. Adherent bacteria were harvested by using 0.5% Triton X-100 solution (Sigma-Aldrich) and centrifuged at 10,000× *g* for 5 min. Viable bacterial cells associated with Caco-2 cells were plated on an MRS agar medium. After incubation at 37 °C for 24 h, colony counts were performed. The binding rate was expressed as a percentage using Equation (2) [27], where A1 is the number of initial bacterial cells (CFU/mL) added to the well and A2 is the number of adherent bacterial cells (CFU/mL).

% adherence = (logA2/logA1) × 100(2)

The inhibition of *E. coli* and *S. Typhi* adhesion by *E. faecium* OV3-6 were performed using a modified method [24]. The bacterial cell and CFS of *E. faecium* OV3-6 were prepared. *E. faecium* OV3-6 was cultured in an MRS broth medium at 37 °C for 18 h and centrifuged at 10,000× *g* for 5 min to separate the bacterial cell and the supernatant. 200 µL of CFS or 10^7^ CFU/mL of *E. faecium* OV3-6 were inoculated at 37 °C with pathogenic bacteria (10^7^ CFU/mL) as co-incubation or 1 h pre-incubated before pathogenic bacteria or 1 h post-incubated after pathogen. After incubation, adherent bacteria were harvested with 0.5% Triton X-100 solution, centrifuged at 10,000× *g* for 5 min and cultured on selective media for 24 h at 37 °C. The percentage inhibition of pathogen adhesion was expressed using the following Equation (3) [24], where T1 is the number of adherent pathogenic bacteria cells (CFU/mL) in the presence of probiotic and T2 is the number of adherent pathogenic bacteria cells (CFU/mL) in the absence of probiotic.

Inhibition of adhesion (%) = (1 − (T1/T2)) × 100(3)

### 2.4. Immunomodulation Associated with Inflammatory Cytokines

The cytokine assay was modified to that carried out in previous studies [28]. Briefly, monolayer Caco-2 cells were co-cultured with 1 ng/mL of lipopolysaccharide (LPS), *E. faecium* OV3-6, enteropathogenic *E. coli*, *S. Typhi*, and *S. Typhimurium* (10^7^ CFU/mL). After incubation at 37 °C in a 5% CO_2_ and 95% air atmosphere for 24 h, the CFS was collected by centrifugation. The cytokines IL-6, IL-12, and IL-10 concentration in the CFS were evaluated by enzyme-linked immunosorbent assay (ELISA) using a commercially available immunoassay kit (Quantikine by R&D Systems, Minneapolis, MN, USA). Caco-2 cells without treatment are used as a negative control.

### 2.5. Antimicrobial Activity against Pathogenic Bacteria

The antimicrobial activity of *E. faecium* OV3-6 was performed by agar well diffusion assay as a modified method from previous studies [28]. *E. faecium* OV3-6 was grown in MRS broth at 37 °C for 18 h. The CFS was obtained by centrifugation at 4 °C at 10,000× *g* for 5 min. A 40 µL of CFS was added to the well (5 mm diameter) into 20 mL appropriate soft agar medium (1% agar) containing a final concentration 10^6^ CFU/mL of the tested strain. After incubation at the optimal growth condition of tested strains, the clear zones size around the wells containing CFS was observed.

The CFS was neutralized, treated with 1 mg/mL catalase (Sigma-Aldrich) and 1 mg/mL proteinase K (Sigma-Aldrich) for 1 h at 37 °C to eliminate the effect of acidic, hydrogen peroxide, and active protein, respectively. The antimicrobial activity against *S. aureus* CIP 76.25 as a sensitive tested strain of treated CFS was tested by agar well diffusion assay. The antimicrobial activity was expressed in arbitrary units per milliliter (AU/mL). An arbitrary unit (AU) was defined as 40 μL of the highest dilution of the twofold serial dilution, which showed a minimal visible inhibition zone against the sensitive cells of tested strain following in Equation (4) [29], where D is the dilution factor, and V is the volume of CFS (µL).

AU/mL = (1000/V) × D(4)

### 2.6. Confirmation of Bacteriocin-Encoding Gene

The detection of bacteriocin-encoding genes in *E. faecium* OV3-6 was evaluated by polymerase chain reaction (PCR). The primer and amplification condition using in this study was described in Table S1. The target genes were genus *Enterococcus*, *entA*, *entB*, *entP*, *entL50A*, *entL50B*, *ent31*, *entQ*, and *entAS48* [11,30]. The obtained DNA template and PCR condition were described above (Section 2.2.5).

### 2.7. Partial Purification of AMPs

*E. faecium* OV3-6 was cultured in 1000 mL of the MRS broth medium at 37 °C for 24 h. The cells were removed by centrifugation (10,000× *g*, 4 °C for 10 min). The supernatant was filtered through the filter (0.22 µm pore size) and assayed for AMPs activity against *S. aureus* CIP 76.25 as tested strain by agar well diffusion assay following in Equation (3) [29].

The supernatant was purified by gently adding ammonium sulfate (RCI-Labscan, Bangkok, Thailand) to obtain 70% saturation and stirred overnight (maintained at 4 °C). The saturated supernatant was centrifuged at 12,000× *g*, 4 °C for 30 min. The pellet was resuspended in 20 mM potassium phosphate buffer (20 mM K_2_HPO_4_, 20 mM KH_2_PO_4_, pH7) [31] before being assayed for AMP activity by an agar well diffusion assay as described above. The crude proteinaceous solution was further fractionated by size using Amicon^®^ Ultra centrifugal filter devices (Merck Millipore, Darmstadt, Germany) with a different molecular weight cut-off (MWCO) which is 50,000, 30,000, and 10,000 MWCO. The AMPs activity and protein concentration were performed in each step of purification. The final AMPs fraction was maintained at −20 °C and subsequently used for further characterization [32].

### 2.8. Molecular Mass and Amino Acid Sequence Determination of AMPs

The molecular weight determination of AMPs was performed using sodium dodecyl sulfate-polyacrylamide gel electrophoresis (SDS-PAGE) according to the method described by Laemmli [33]. Briefly, the partially purified AMPs were resolved in SDS-PAGE with 12% (*v*/*v*) separating gel on the Mini-PROTEAN Tetra Cell (Bio-Rad, CA, USA). A 20 µL of partially purified AMPs sample (2.45 mg protein/mL) was mixed with 20 µL 2× sample buffer (12.5%, *v*/*v* of 0.5 M Tris–HCl pH 6.8, 25%, *v*/*v* of 25% glycerol, 20%, *v*/*v* of 10% SDS, 36.5%, *v*/*v* distilled water, 1%, *v*/*v* of 1% bromophenol blue, 5%, *v*/*v* β-mercaptoethanol). A 10 µL of the mixed sample was loaded into the gel. A low molecular weight protein marker (MW 1700 to 42,000 Da) (Cell-Signaling Technology, Beverly, MA, USA) was used to compare the protein’s molecular weight. After electrophoresis, the gels were stained with coomassie blue G-250 stain solution for 2 h and destained until the stain background disappeared. The gels were washed in water for 1.5 h to remove the stain solution from the gels and sliced into 4 pieces [22]. One of the gels was overlaid with 20 mL BHI soft agar containing *S. aureus* CIP 76.25 (10^6^ CFU/mL). The inhibition zone was observed after incubation at 37 °C for 24 h. The other gels were used for further determination of molecular mass and amino acid sequence.

The AMPs bands from SDS- PAGE gel were trypsin digested and peptides extracted according to standard techniques [34]. Peptides were analyzed by using liquid chromatograph-mass spectrometry/mass spectrometry (LC-MS/MS) instrument with electrospray ionization mass spectrometer of the Shimadzu Prominence nano HPLC system (Shimadzu, Tokyo, Japan) coupled to a 5600 TripleTOF mass spectrometer (AB SCIEX, Concord, ON, Canada). Tryptic peptides were loaded onto an Agilent Zorbax 300SB-C18, 3.5 μm (Agilent Technologies, DE, Waldbronn, Germany) and separated with a linear gradient of water/acetonitrile/0.1% formic acid (*v*/*v*). Spectra were analyzed to identify interest proteins using Mascot sequence matching software (Matrix Science, London, UK) with the MSPnr100 database.

### 2.9. Enzyme Sensitivity, Physicochemical Treatments and Long-Term Storage of AMPs

The effect of enzymes on partially purified AMPs was determined by incubating the AMPs with catalase, α-chymotrypsin, trypsin, proteinase K (Sigma-Aldrich) with 1 mg/mL of final enzyme concentration for 1 h incubation at 37 °C and then the reactions were stopped by boiling for 3 min [35]. Thermal sensitivity evaluation of partially purified AMPs was performed by keeping partially purified AMPs at 0, 4, 37, 45, 60, and 100 °C for 0, 15, 30, 60, 90, and 120 min, and autoclaved at 121 °C for 15 min [31]. The partially purified AMPs were adjusted from pH 2.0 to 12.0 with 1 N HCl or 1 N NaOH to determine the effect of pH on partially purified AMPs. After 1 h of incubation at room temperature, the pH of samples was neutralized [36]. The effect of NaCl on partially purified AMPs was assayed by adding 10 and 30% NaCl to the samples. The treated samples were stored at 4 °C for 1 month. Every week, the samples were taken to determine antimicrobial activity [22]. The partially purified AMPs were stored at −20, 4, 37 °C and room temperature for 3 months in long-term storage. The samples were collected to determine the antimicrobial activity [22]. A 1 mg/mL nisin as a commercial preservative was used as a positive control. The antimicrobial activity against *S. aureus* CIP 76.25 of each treated partially purified AMPs and nisin was assayed using the agar well diffusion assay.

### 2.10. Statistical Analysis

All experiments were performed at least three replicates, and values are presented as mean ± standard deviations. Data were conducted using SPSS software version 17.0 (SPSS Inc., Chicago, IL, USA). Significant differences between means were statistically tested using for one-way analysis of variance (ANOVA) with Duncan’s post hoc test. Differences were considered significant at *p* < 0.05.

## 3. Results

### 3.1. Probiotic Properties and Safety Assessment

*E. faecium* OV3-6 tolerated to simulated gastric juice (Figure S1a). The bacteria viability was unaffected in gastric juice (*p* < 0.05) during 1 h with survival abilities of 98.48%. While at 2, 3, and 4 h of incubation, the percentage survival of the bacteria was decreased to 90.29, 88.45, and 88.16%, respectively. Moreover, the strain OV3-6 showed resistance to simulated small intestine conditions with percentage survival of the strain during 1 and 2 h were 99.60, 99.49, respectively. However, their viability slightly decreased at 4 and 6 h exposure was 96.89 and 94.33%, respectively (Figure S1b).

*E. faecium* OV3-6 showed the BSH activity with precipitation zone surrounding colonies. As safety claim, the strain showed blood α-hemolysis or absence of hemolysis (data not shown). In the antibiotic susceptibility tests, the OV3-6 was sensitive to eight antibiotics, including ampicillin, chloramphenicol, clindamycin, gentamicin, penicillin G, streptomycin, tetracycline, and vancomycin. However, the strain showed intermediate sensitivity to erythromycin and kanamycin.

**Figure 1:**
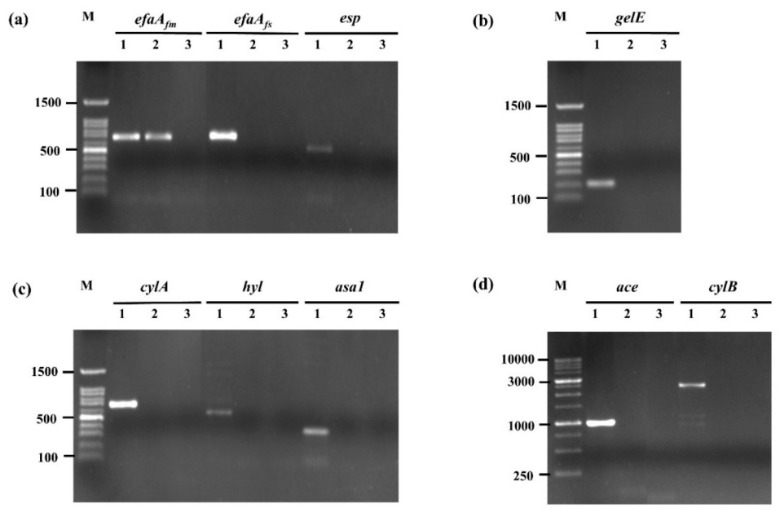
Amplification of virulence gene in *E. faecium* OV3-6 by PCR. (**a**) *efaA_fm_*, *efaA_fs_*, and *esp* genes; (**b**) *gelE* gene; (**c**) *cylA*, *hyl*, and *asa1* genes; (**d**) *ace* and *cylB* genes; Lane M: 100 bp and 1 Kb DNA markers; Lanes 1: products of positive controls; Lanes 2: products of *E. faecium* OV3-6; Lanes 3: products of negative controls.

The safety of *E. faecium* OV3-6 was investigated by the screening of genes encoding different virulent factors. PCR performed the presence of virulence genes encoding *ace*, *asa1*, *cylA*, *cylB*, *efaAfs*, *esp*, *hyl*, and *gelE*. The agarose gel electrophoresis revealed that *E. faecium* OV3-6 does not harbor any of these virulence genes except *efaA_fm_*_,_ which showed 735 bp of amplification product (Figure 1).

### 3.2. Inhibition of Adhesion on Caco-2 Cell Model

The adhesion rate of *E. faecium* OV3-6, *E. coli*, and *S*. Typhi to Caco-2 cells were 89.76, 69.12, and 92.15%, respectively. All bacteria are a significant difference in adhesion rate.

**Figure 2:**
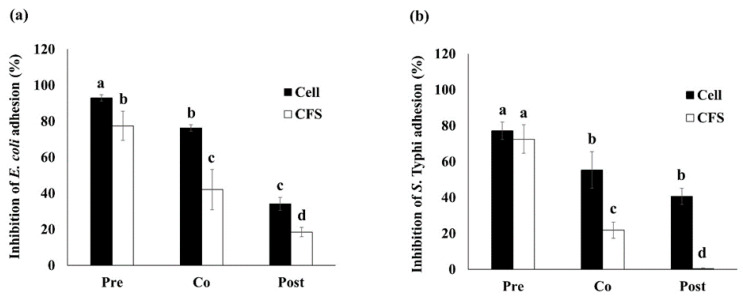
The inhibition of *E. coli* (**a**) and *S. Typhi* (**b**) adhesion to Caco-2 cell by bacteria cell and CFS of *E. faecium* OV3-6. Three conditions: *E. faecium* OV3-6 was treated on Caco-2 cell at 1 h before pathogenic bacteria was added as pre-incubation (Pre); *E. faecium* OV3-6 and pathogenic bacteria were added at the same time as co-incubation (Co); *E. faecium* OV3-6 was treated on Caco-2 cell at 1 h after pathogenic bacteria was added as post-incubation (Post). Data are shown as means ± SD of triplicate experiments. The different letters above columns indicate that the values are significantly different (*p* < 0.05).

In pre-incubation, the inhibition of *E. coli* adhesion to Caco-2 cells by living cell and CFS of *E. faecium* OV3-6 showed a significant decrease of 92.92 and 77.37%, respectively. The co-incubation showed 76.25 and 42.08%, respectively. And the post-incubation showed 34.17 and 18.33%, respectively (Figure 2a).

In pre-incubation, the inhibition of *S*. Typhi adhesion to Caco-2 cells by living cells and CFS of *E. faecium* OV3-6 showed 77.19 and 72.50%, respectively (Figure 2b). The co-incubation showed a significant decrease with 55.31 and 21.87%, respectively. And the post-incubation showed 40.62 and 0.31%, respectively.

### 3.3. Immunomodulation Associated with Inflammatory Cytokines

The LPS significantly (*p* < 0.05) induced pro-inflammatory cytokine IL-6 secretion (45.30 ± 2.54 pg/mL) higher than any treatments (Table 1). The *E. faecium* OV3-6 significantly induced IL-6 secretion (12.36 ± 2.80 pg/mL) less than any treatments except untreated. *S. Typhi*, LPS, and *E. coli* significantly induced pro-inflammatory cytokine IL-12 secretion of 6.38 ± 2.06, 6.04 ± 0.52, and 5.59 ± 1.38 pg/mL, respectively. The *E. faecium* OV3-6 and the co-culture *E. faecium* OV3-6 with each pathogen or LPS significantly induced IL-6 and IL-12 secretion less than only LPS and each pathogen. Each treatment increased anti-inflammatory cytokine IL-10 secretion when compared with untreated. *E. faecium* OV3-6 significantly induced IL-10 secretion (247.59 ± 17.43 pg/mL) higher than any treatments.

### 3.4. Antimicrobial Activity against Pathogenic Bacteria

The antimicrobial activity of *E. faecium* OV3-6 was determined as the inhibition zone by the agar well diffusion assay summarized in Table 2. The CFS of *E. faecium* OV3-6 showed antimicrobial activity against tested strains such as *B. cereus* ATCC 11778, *C. maltaromaticum* NCDO 2760, *L. innocua* CIP 80.11^T^, *L. ivanovii* SLCC 2379, *S. aureus* CIP 76.25, and *S. mutans* ATCC 25175.

The verification of AMPs activity in CFS was assessed by agar well diffusion assay. The neutralized CFS was treated with catalase to remove the hydrogen peroxide effect and proteinase K to confirm the effect of protein. The result showed the treated CFS had antimicrobial activity against *S. aureus* CIP 76.25 (400 AU/mL). However, the proteinase K causes the CFS to lose the inhibition activity.

### 3.5. Confirmation of Bacteriocin-Encoding Gene

**Figure 3:**
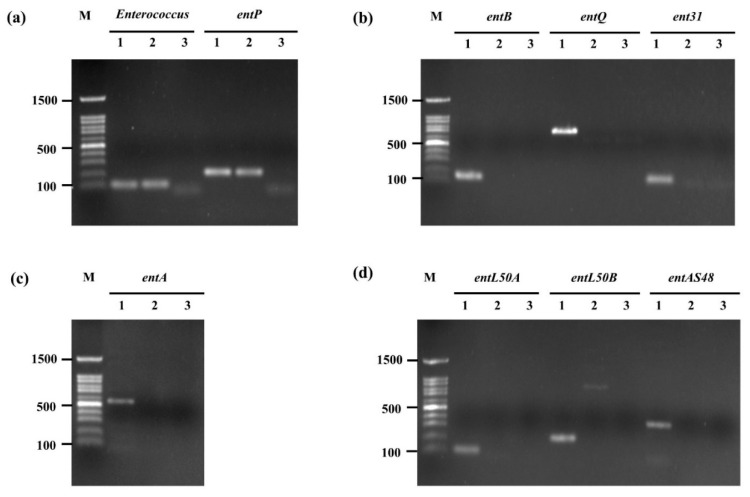
Amplification of genus *Enterococcus* gene and bacteriocin genes in *E. faecium* OV3-6 by PCR. (**a**) Genus *Enterococcus* and *entP* genes; (**b**) *entB*, *entQ*, and *ent31* genes; (**c**) *entA* gene; (**d**) *entL50A*, *entL50B*, and *entAS48* genes; Lane M: 100 bp DNA markers; Lanes 1: products of positive controls; Lanes 2: products of *E. faecium* OV3-6; Lanes 3: products of negative controls.

To confirm the presence of the genus *Enterococcus* spp. gene (*ent*) and detection of bacteriocin-encoding genes *entP*, *ent50A*, *ent50B*, *entAs48*, *entA*, *entB*, *entQ*, and *ent31* in *E. faecium* OV3-6 were evaluated by PCR. Results from agarose gel electrophoresis revealed that *E. faecium* OV3-6 did not harbor any bacteriocin genes except *entP*, which showed 216 bp of amplification product (Figure 3). The amplification of *E. faecium* OV3-6 presented of the *Enterococcus* gene (112 bp) indicated this strain was genus *Enterococcus* (Figure 3a).

### 3.6. Partial Purification of AMPs

The total protein content in 1000 mL CFS produced by *E. faecium* OV3-6 was 1260.00 mg and 400,000 AU of activity. The CFS was purified by two steps, as shown in Table 3. The first step was 70% saturation of ammonium sulfate precipitation, which increased the specific activity against strain *S. aureus* CIP 76.25 by 5.57-folds. The step of the molecular weight cut-off by using Amicon^®^ Ultra centrifugal filter increased the specific activity and the increase in specific activity by 5224.49 AU/mg and 16.46-folds, respectively.

### 3.7. Molecular Mass and Amino Acid Sequence Determination of AMPs

**Figure 4:**
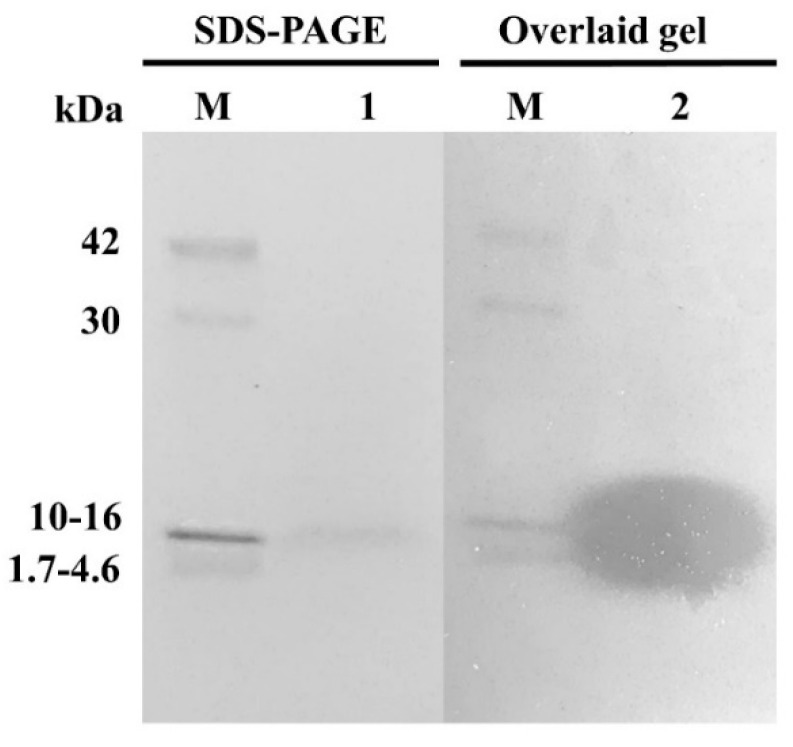
SDS-PAGE of the partially purified AMPs produced by *E. faecium* OV3-6. Lane M: low molecular weight protein marker (MW 1700–42,000 Da); Lane 1: SDS-PAGE band; Lane 2: inhibition zone of the SDS-PAGE band overlaid with tested strain *S. aureus* CIP 76.25 in BHI agar.

The partially purified AMPs were separated in SDS-PAGE. The result showed a clear single protein band, and molecular weight was identified at approximately 10–16 kDa (Figure 4). After the partially purified AMPs band from SDS-PAGE was overlaid with *S. aureus* CIP 76.25, the antimicrobial activity against indicator strain has appeared as an inhibition zone.

The amino acid sequence of partially purified AMPs was retrieved from the Mascot Server. The data showed amino acid sequence from ~10–16 kDa protein band were matched (40%) with 50S ribosomal proteins L29 of *Enterococcus faecalis*, which molecular masses of 7.325 kDa and isoelectric point (pI) value of 9.60. This protein matched with the LC-MS/MS spectrum assigned the amino acid sequence ELTTAEMLDKEK and FQLATGQLENTAR (Figure S2).

### 3.8. Enzyme Sensitivity, Physicochemical Treatments and Long-Term Storage of AMPs

The effects of enzymes, temperature pH, and NaCl on the antimicrobial activity of partially purified AMPs produced by *E. faecium* OV3-6 (PPA OV3-6) and 1 mg/mL nisin are shown in Table S2. PPA OV3-6 and nisin were resistant to catalase but completely inactivated by the proteinase enzyme (α-chymotrypsin, trypsin, and proteinase K). After treatment with various temperatures, PPA OV3-6 was heat-stable at temperatures 4, 37, 45, 60 °C for120 min and 100 °C for 15 min. The activity of PPA OV3-6 and nisin were gradually decreased after heating at 100 °C for 30–120 min. At autoclave condition, the activity was decreased 25% from the beginning, but nisin completely disappeared. PPA OV3-6 was found fully active after treatment with pH 2–10, but at pH 12, the activity was lost 50%. In the case of nisin, the activity was stable at pH 2–7 but decreased after treatment with pH 8–12. The 10–30% (*w*/*v*) of NaCl did not affect both tested substances. In long-term storage, the antimicrobial activity of PPA OV3-6 remained full activity at −20 and 4 °C till 3 months and at room temperature (RT) after a week of storage. However, 1 mg/mL nisin at 4 °C was decreased after three weeks and at RT after a week. At 37 °C of storage, both tested substances lost 50% of activity within one week and still gradually decreased until the end of three months of storage.

## 4. Discussion

*Enterococcus* genus has been an object of increasing scientific work because of its wide range of health-promoting effects for a probiotic [26]. Probiotic *E. faecium* OV3-6 exhibited survival abilities in simulated gastric juice for 4 h (88.16%). Similar to the observations of genus *Enterococcus* isolated from food sources, Maia reported that *E. faecium* showed percentage survival between 86–107% in pH 3 for 4 h [37]. The proton pumps such as the F_1_F_0_-ATPase [38] or utilized by the glutamate decarboxylase (GAD) system [39] are important mechanisms involved in the acid resistance regulation of LAB under acidic conditions. Another mechanism is alkali production by the arginine deaminase (ADI) or urease system that increases the cell’s internal pH [40]. The OV3-6 also showed high survival (94.33%) in 0.3% bile salts and 1 mg/mL pancreatin for 6 h of exposure (Figure S1b). The selected *E. faecium* strains were more resistant to bile salt solution [41,42]. The resistance to bile salt of some LAB strains was also related to BSH enzyme activity, which can hydrolyze combined bile salt and thus reduce their toxic and side effects [43]. This study suggested that *E. faecium* OV3-6 was more tolerant to the damaging effect of GI tract. Furthermore, the OV3-6 presents positive BSH activity. Our results are consistent with a report by Zhang et al. that found that *E. faecium* WEFA23, WEFA24, and WEFA30 were efficient in hydrolyzing taurodeoxycholic acid and precipitated out deoxycholate on medium [42]. Normally, the conjugated bile acids were hydrolyzed by the BSH enzyme to unconjugated bile acids. The unconjugated products are precipitated at the low pH caused by fermentation by LAB. BSH increases the survival and persistence of producing strains in intestinal transit [44].

The hemolytic activity of *E. faecium* OV3-6 was evaluated to consider a safety prerequisite for selecting a probiotic strain. The strain displayed α-hemolysis, indicating that this strain was no hydrolysis of a red blood cell. This result is consistent with the study of Vandera and İspirli [45,46]. However, some strains of *Enterococcus* spp. isolated from equipment surfaces, raw materials, and traditional cheeses express γ-hemolysis and β-hemolysis [47].

Antibiotic resistance in enterococci is a major public health concern. Because these organisms are microbial flora in the GI tract of humans and warm-blooded animals [48], antibiotic-resistant genes might be transferred to opportunistic bacteria during passage in the GI tract via chromosomes, plasmid, or transposons [21]. The *E. faecium* OV3-6 was sensitive to most clinically relevant antibiotics but only had intermediate sensitivity to erythromycin and kanamycin. This finding is consistent with the previous reports [49,50]. They suggest that *E. faecium* is expressed moderately susceptible to erythromycin and kanamycin. Intermediate sensitivity indicates that the microbial strain is under the resistant category. *E. faecium* can be considered a very poor donor in terms of the erythromycin or kanamycin resistance genes transfer to other enterococci [51]. Applying this strain in vivo by administering may be sensible at the highest possible dose of the antibiotic. However, the candidate probiotic must be more characterized to ensure the absence of antibiotic resistance gene in the genome level in further study.

Enterococci can express genes encoding some virulence factors that may enhance their pathogenicity. Therefore, the virulence factors of *E. faecium* OV3-6 were investigated to confirm the safety of probiotics. The result shows the only *efaA_fm_* (cell wall adhesion in *E. faecium*) was detected. Our findings are consistent with some reports that *efaA_fm_* gene was present in *E. faecium* BP8 and *E. faecium* EYT17 [46,52]. Abouelnaga et al. suggested that the *efaA_fm_* gene was found in various enterococci species isolated from both fermented and unfermented foods. It is possible to explain the distribution of the *efaA_fm_* gene given the nature of its association with *E. faecium* [53]. However, the *efaA_fm_* gene may play a beneficial role for probiotic bacteria. They are presumed to be involved in mechanisms by which the enterococcal cells adhere to biotic and abiotic surfaces [54].

Adherence to the host intestinal surface is considered a principal criterion to exhibit probiotic functional properties. In our study, the capacity of *E. faecium* OV3-6 showed 89.67% adhesion rate to the Caco-2 cell, which is a strong adherence range greater than previously published studies [49,55]. This result is not surprising about adherence capable of *Enterococcus* to gut epithelial cells because they are a common part of the indigenous human microbiota, especially in the GI tract [6].

Adhesion is the main mechanism of pathogenic bacteria causing infections in humans and animals. The bacteria involved in severe gastric infections are *H. pylori*, *C. difficile*, *E. coli*, *L. monocytogenes*, and *Salmonella* [8]. The living cell of *E. faecium* OV3-6 reduced the adhesion of *E. coli* and *S. Typhi* to Caco-2 cells greater than CFS (Figure 2). One possible explanation for adhesion inhibition in this study is the different proteins located on the bacterial cell wall, which are specific to a receptor on the intestinal epithelial cell of the host [56]. Also, the auto-aggregation and co-aggregation of cells are related to the competition between probiotics and pathogens for adhesion on host tissue [57,58]. The secretion of organic acid of probiotics such as lactic and acetic acids related to the intracellular pH lowering or their intracellular accumulation can cause pathogens’ death. In addition, probiotics have been reported to produce bacteriocins that displayed an inhibitory against pathogen bacteria [59].

Immunomodulatory properties play an important role in the mode of action of probiotics. LPS is the major initiator of intestinal epithelial cell (IEC) inflammation and potent immunomodulatory components derived from the cell wall of Gram-negative bacteria such as *E. coli* and *Salmonella* [60,61]. The immune recognition of LPS is mediated through the toll-like receptor-4 (TLR4) receptor complex in epithelial cells of the intestine and immune cell types, predominantly macrophages, B cells, and dendritic cells [62]. Activation of innate immune cells by LPS via TLR4 initiates the secretion of pro-inflammatory cytokines such as TNF-α, IL-1β, IL-6, IL-12, and IFN-γ [63]. IL-10 is potent immunoregulatory cytokine and anti-inflammatory properties produced by activated macrophages and T cells. IL-10 suppresses T cell proliferation and the release and function of many pro-inflammatory cytokines, such as IL-1 and IL-6. Probiotic bacteria may regulate the expression of this cytokine [64]. This study showed that *E. faecium* OV3-6 suppressed the production of pro-inflammatory cytokines IL-6, IL-12 comparing with pathogens and LPS alone. The OV3-6 also augmented the production of anti-inflammatory cytokines IL-10, contributing to intestinal innate immunity and homeostasis regulation. Our results agree with studies of other authors in the macrophage cell line [65] and human enterocyte-like HT-29 cells model [66]. In contrast with *E. faecium* FC-K, it could induce secretion of pro-inflammatory cytokine TNF-α, IL-6, and IL-12 from mouse peritoneal macrophage [67].

CFS’s antimicrobial activities produced by *E. faecium* OV3-6 were active in a narrow spectrum against Gram-positive bacteria but not against Gram-negative bacteria and yeast (Table 2). The various antimicrobial substances secreted in CFS of LAB include organic acids (such as lactic acid, acetic acid, and propionic acid) [68,69], hydrogen peroxide, bacteriocins [59], and other compounds such as some RPs [15,70]. Most of the bacteriocin show inhibit the growth of closely related species [71]. According to the result of this study that *E. faecium* OV3-6 shows antimicrobial activity against only Gram-positive bacteria. In contrast with the report of Vasilchenko et al. that bacteriocin, namely Enterocin-7, showed a broad spectrum against both Gram-positive and Gram-negative bacteria [72]. This study suggests that some antimicrobial substances secreted by *E. faecium* OV3-6 are proteinaceous compounds as AMPs because activity was not changed after pH neutralization and treatment with catalase enzyme and after treatment with a proteolytic enzyme, the antimicrobial activity disappeared.

Interestingly, one of the most identified AMPs produced by LAB is bacteriocins. In the present study, *E. faecium* OV3-6 has been shown carrying out only the *entP* gene (Figure 3). The bacteriocin-encoding genes *entP* have been found in Enterococci bacteria, especially in *E. faecium* [73,74]. The *entP* encodes a 71-amino-acid prepeptide consisting of a 44-aminoacid mature bacteriocin and a 27-amino-acid signal peptide [75]. Enterocin P is a class IIa bacteriocins with a very strong anti-listerial effect and a wide range of spoilage and foodborne Gram-positive pathogenic bacteria. Nevertheless, bacteriocin structural gene presence does not necessarily imply the capacity to produce it [76]. It is important to purify and characterize these bacteriocins from *E. faecium* OV3-6.

The characterization of the active proteinaceous compounds of *E. faecium* OV3-6 after partial purification and identification by a proteomic analysis base on SDS-PAGE, we found only one significant protein band belongs to a mass ~10–16 kDa (Figure 4). Amino acid sequence from mass spectrometry (LC-MS/MS) analysis has allowed us to identify the active proteinaceous compounds of *E. faecium* OV3-6 reveals the 50S ribosomal proteins L29. The antimicrobial activity of the 50S ribosomal proteins L29 from *E. faecium* is unprecedented. Ribosomal protein L29 is a component of a large 50S subunit that also possesses antimicrobial activity [14]. This result is in agreement with a few reported secreted RPs identified in other bacteria species. In particular, 30S ribosomal proteins S19, S20, and S21; 50S ribosomal proteins L24 and L29 have been shown in the CFS of *Lactobacillus sakei* subsp. *Sakei* 2a were active against the *Listeria* spp., *Enterococcus* spp., *S. epidermidis*, and *L. sakei* strains [70,77]. Also, 50S ribosomal proteins L29 of *Paenibacillus polymyxa* Kp10 were antimicrobial proteins against *L. monocytogenes* [78]. The antimicrobial mechanisms of RPs have been described by Carvalho that secreted RPs of *L. sakei* subsp. *sakei* 2a decreased the membrane potential (ΔΨ) and increased the ATP efflux in *L. monocytogenes* [77]. The decrease in ΔΨ could be involved several degrees of membrane disruption. The modification of ΔΨ has been described already with other AMPs class IIa bacteriocins like enterocin P [79]. The high isoelectric point of RPs may indicate a high affinity to a negatively charged phosphate group of nucleic acids and bacteria cell membrane. However, this remains to be elucidated [78]. Generally, AMPs are amphiphilic and cationic which means the net charge at neutral pH varies from +2 to +9. The polycationic peptides could also attract toward the anionic cell membrane of targeted bacteria via electrostatic interaction. There caused structural microbial membrane disruptions, leading to cell lysis and death [80,81].

50S ribosomal proteins L29 are mainly active AMPs of our *E. faecium* OV3-6. Interestingly, these partials purify AMPs also maintain their antimicrobial activity at a wide range of pH between 2 to 10. And after various temperature treatments (4 to 100 °C) for 15 min, their activity did not change, and the activity still appears under autoclaving. The AMPs from OV3-6 were heat-stable. At storage conditions for 12 weeks, we found partial purified AMPs retains its activity at deep freezer and refrigerator condition. A similar study has reported 50S ribosomal proteins L27 and L30 from *P. polymyxa* Kp10 [15]. The properties of 50S ribosomal proteins L29 secreted by *E. faecium* OV3-6 look like the characterized bacteriocin of other *Enterococcus* under the effect of the enzyme, physicochemical treatments, and stability conditions which showed in many reports. Bacteriocin produced by *E. faecalis* KT11 against some pathogenic bacteria remained stable at pH values ranging from 2 to 11 and after autoclaving at 121 °C for 30 min [82]. The antimicrobial activity of two bacteriocins (bacALP7 and bacALP57) from *E. faecium* was stable over a wide range of pH and temperatures [36]. The AMPs produced by *E. faecium* OV3-6 are suitable to be applied as a bio-preservative agent in functional foods and beverages. Although, nisin is currently the only bacteriocin widely used as a food preservative approved by US-FDA [12]. This study indicates that AMPs of *E. faecium* OV3-6 are more resistant to a wide range of pH than nisin, especially in the alkaline range (pH 8–12). In addition, it is also more heat-stable under autoclave conditions than nisin. These AMPs may be an alternative natural preservative from probiotics, which have qualifications equivalent to or more than nisin. In further steps, the AMPs will be studied about the mode of action to understand deeply in the animal model. This will make it easy and suitable to be applied as a biopreservative in food.

## 5. Conclusions

Overall, this study demonstrated that *E. faecium* OV3-6 isolated from fermented (*Houttuynia cordata* Thunb.) beverage has promising probiotic potential. The outcomes of this work claim that our strain is resistant to simulated GI conditions. The safety assessment revealed that this strain was susceptible to most clinically relevant antibiotics, especially vancomycin, and does not harbor the virulence genes for *ace*, *asa1*, *cylA*, *cylB*, *efaA_fs_*, *esp*, *hyl*, and *gelE*. This strain also presents high adhesion capacity and anti-pathogenic bacterial adhesion on the Caco-2 cell. Additionally, it shows immune-modulatory activity, reducing pro-inflammatory cytokines IL-6 and IL-12 secretion in the Caco-2 cell and inducing the secretion of anti-inflammatory cytokines IL-10. This study is the first report showing 50S ribosomal proteins L29 secreted from *E. faecium* OV3-6 having bactericidal activity. The active protein exhibits heat-stable and stability over a wide pH range. It may be considered as an interesting candidate for future use as a probiotic and bioprotective for application in the food or feed industries. However, in vivo on animal models and clinical trials are essential to authenticate the safety of *E. faecium* OV3-6 before it can be incorporated into the human food chain.

## Figures and Tables

**Table 1:** Secretion of cytokines by Caco-2 when treated with LPS, *E. coli*, *S. Typhi*, *S. Typhimurium*, and *E. faecium* OV3-6.

Treatments	Secretion of Cytokines (pg/mL)		
	IL-6	IL-12	IL-10
Untreated	5.67 ± 0.41 ^f^	0.34 ± 0.03 ^e^	8.32 ± 1.33 ^f^
LPS	45.30 ± 2.54 ^a^	6.04 ± 0.52 ^a^	99.84 ± 4.05 ^e^
*E. coli*	39.27 ± 4.00 ^b^	5.59 ± 1.38 ^a^	112.20 ± 1.20 ^d,e^
*S. Typhi*	39.92 ± 3.56 ^b^	6.38 ± 1.06 ^a^	97.20 ± 2.80 ^e^
*S. Typhimurium*	37.00 ± 0.61 ^b^	4.03 ± 1.00 ^b^	126.18 ± 15.10 ^c,d^
*E. faecium* OV3-6	12.36 ± 2.80 ^e^	1.80 ± 0.31 ^d^	247.59 ± 17.43 ^a^
LPS *+ E. faecium* OV3-6	31.74 ± 2.29 ^c^	3.57 ± 0.30 ^b,c^	110.02 ± 2.24 ^d,e^
*E. coli + E. faecium* OV3-6	26.58 ± 5.48 ^c,d^	2.45 ± 0.87 ^c,d^	130.57 ± 10.30 ^c^
*S. Typhi+ E. faecium* OV3-6	25.42 ± 2.86 ^d^	2.71 ± 0.37 ^b,c,d^	137.44 ± 2.80 ^c^
*S. Typhimurium+ E. faecium* OV3-6	22.80 ± 2.73 ^d^	1.97 ± 0.42 ^c,d^	156.11 ± 16.18 ^b^

Data are shown as means ± SD of triplicate experiments. Means of individual trials within a column with the different letters are significantly different (*p* < 0.05) with an individual column.

**Table 2:** The antimicrobial activity of CFS produced by *E. faecium* OV3-6.

Tested Strains	Inhibition Zone (mm)
**Gram-positive bacteria**	
*Bacillus cereus* ATCC 11778	7.00 ± 0.10 ^d^
*Brochothrix thermosphacta* DSMZ 20171^T^	NI
*Carnobacterium maltaromaticum* NCDO 2760	14.70 ± 0.24 ^b^
*Clostridium perfringens*	NI
*Listeria innocua* CIP 80.11^T^	12.05 ± 0.65 ^c^
*Listeria ivanovii* SLCC 2379	14.60 ± 0.30 ^b^
*Propionibacterium acnes* ATCC 6919	NI
*Staphylococcus aureus* ATCC 5923	NI
*Staphylococcus aureus* CIP 76.25	15.63 ± 0.03 ^a^
*Staphylococcus epidermidis* ATCC 12228	NI
*Streptococcus mutans* ATCC 25175	6.25 ± 0.25 ^e^
**Gram-negative bacteria**	
*Cronobacter sakazakii* ATCC BAA-894	NI
*Escherichia coli* ATCC 5922	NI
*Escherichia coli* CIP 76.24	NI
*Pseudomonas aeruginosa* ATCC 27853	NI
*Salmonella* Typhi DMST 22842	NI
*Salmonella* Typhimurium TISTR 1469	NI
*Shigella sonnei*	NI
*Vibrio harveyi* ATCC BAA-1119	NI
**Yeast**	
*Candida albicans* ATCC 90028	NI

Data are shown as means ± SD of triplicate determinations. The different letters indicate that the values are significantly different (*p* < 0.05). The inhibition zone includes diameters of agar well (5 mm of diameter). No inhibition (NI).

**Table 3:** Purification steps of AMPs produced by *E. faecium* OV3-6.

Step of Purification	Total Volume (mL)	Activity (AU/mL)	Protein (mg/mL)	Total Activity (AU)	Total Protein (mg)	Specific Activity (AU/mg)	Yield (%)	Increase in Specific Activity (Fold)
Cell-free supernatant	1000	400	1.26	400,000	1260.00	317.46	100.00	1.00
Crude (NH_4_)_2_SO_4_ precipitation	100	3200	1.81	320,000	181.00	1767.96	80	5.57
Molecular weight cut off	24	12,800	2.54	307,200	58.80	5224.49	76.80	16.46

## Data Availability

The data are available as the Supplementary Documents.

## Supplementary Materials

The following are available online at https://www.mdpi.com/article/10.3390/foods10102264/s1, Table S1: Primers used for PCR amplification of virulence and bacteriocin genes in *Enterococcus*, Table S2: Enzyme sensitivity, physicochemical treatments, and long-term storage condition on partially purified antimicrobial peptides produced by *E. faecium* OV3-6 (PPA OV3-6) and 1 mg/mL commercial preservative nisin, Figure S1: The percentage survival of *E. faecium* OV3-6 in simulated gastric juice (a) and simulated small intestinal condition (b). Data represent means from three repeats (±SD). The different letters above columns indicate that the values are significantly different (*p* < 0.05), Figure S2: Mass spectrometry spectrum of amino acid sequence ELTTAEMLDKEK and F QLATGQLENTAR from ~10–16 kDa protein band of *E. faecium* OV3-6, which matches 50S ribosomal proteins L29 are shown in bold text.
