# Supplementary material for: Potential Probiotic Enterococcus faecium OV3-6 and Its Bioactive Peptide as Alternative Bio-Preservation
Source: Foods. 2021 Sep 24;10(10):2264. doi: 10.3390/foods10102264 (PMC8534580; doi:10.3390/foods10102264)
Supplement: Supplementary file 1 [file foods-10-02264-s001.zip › foods-1335231-supplementary.pdf]

Table S1. Primers used for PCR amplification of virulence and bacteriocin genes in *Enterococcus*.

Gene	Primer	Sequences (5′–3′)	AT (°C)	Length (bp)	References
genus *Enterococcus*	Ent 1	TACTGACAAACCATTCATGATG	56	112	[30]
	Ent 2	AACTTCGTCACCAACGCGAAC			
asa1	ASA11	GCACGCTATTACGAACTATGA	56	375	[11]
	ASA12	TAAGAAAGAACATCACCACGA			
esp	ESP14F	AGATTTCATCTTTGATTCTTGG	56	510	[11]
	ESP12R	AATTGATTCTTTAGCATCTGG			
ace	ACE-F	GAATTGAGCAAAAGTTCAATCG	56	1008	[11]
	ACE-R	GTCTGTCTTTTCACTTGTTTC			
cylA	CYT I	ACTCGGGGATTGATAGGC	58	688	[11]
	CYT IIb	GCTGCTAAAGCTGCGCTT			
cylB	cylB1	AAGTACACTAGTAGAACTAAGGGA	56	2020	[11]
	cylB2	ACAGTGAACGATATAACTCGCTATT			
$efaA_{fm}$	TE37	AACAGATCCGCATGAATA	54	735	[11]
	TE38	CATTTCATCATCTGATAGTA			
$efaA_{fs}$	TE5	GACAGACCCTCACGAATA	54	705	[22]
	TE6	AGTTCATCATGCTGTAGTA			
gelE	GEL11	TATGACAATGCTTTTTGGGAT	56	213	[23]
	GEL12	AGATGCACCCGAAATAATATA			
hyl	HYL n1	ACAGAAGAGCTGCAGGAAATG	56	276	[23]
	HYL n2	GACTGACGTCCAAGTTTCCAA			
entA	EntAF	GAGATTTATCTCCATAATCT	45	542	[11]

	EntAR	GTACCACTCATAGTGGAA			
entB	EntBF	GAAAATGATCACAGAATGCCTA	41	159	[11]
	EntBR	GTTGCATTTAGAGTATACATTTG			
entP	EntPF	ATGAGAAAAAAATTATTTAGTTT	41	216	[11]
	EntPR	TTAATGTCCCATACCTGCCAAACC			
entL50A	EntL50AF	CCATGGGAGCAATCGCAAAA	50	135	[11]
	EntL50AR	AAGCTTAATGTTTTTTAATCCACTCAAT			
entL50B	EntL50BF	ATGGGAGCAATCGCAAAATTA	49	252	[11]
	EntL50BR	TAGCCATTTTTCAATTTGATC			
ent31	Ent31F	CCTACGTATTACGGAAATGGT	58	130	[11]
	Ent31R	GCCATGTTGTACCCAACCATT			
entQ	EntQF	GGAATAAGAGTAGTGGAATACTGATATGAGAC	60	653	[11]
	EntQR	AAAGACTGCTCTTCCGAGCAGCC			
entAS48	EntAS48F	GAGGAGTATCATGGTTAAAGA	56	339	[11]
	EntAS48R	ATATTGTTAAATTACCAA			

Table S2. Enzyme sensitivity, physicochemical treatments, and long-term storage condition on partially purified antimicrobial peptides produced by *E. faecium* OV3-6 (PPA OV3-6) and 1 mg/mL commercial preservative nisin.

**Treatments**	**Activity (AU/mL)[a]**	
	**PPA OV3-6**	**1 mg/mL nisin**
Untreated (control)	12,800	1600
**Enzyme**		
Catalase	12,800	1600
α-chymotripsin	0	0
Trypsin	0	0
Proteinase K	0	0
**Temperature**		
4 °C / 15–120 min	12,800	1600
37 °C / 15–120 min	12,800	1600
45 °C / 15–120 min	12,800	1600
60 °C / 15–120 min	12,800	1600
100 °C / 15 min	12,800	1600
100 °C / 30min	6400	1600
100 °C / 60min	6400	800
100 °C / 90min	3200	200
100 °C / 120min	1600	100
121 °C / 15 min	3200	0
**pH**		
2–7	12,800	1600
8–10	12,800	800
12	6400	200
**NaCl**		
10% / 1–4 weeks	12,800	1600
30% / 1–4 weeks	12,800	1600
**Long-term storage**		
−20 °C / 1–12 weeks	12,800	1600
4 °C / 1–3 weeks	12,800	1600
4 °C / 4–7 weeks	12,800	800

4 °C / 8–11 weeks	12,800	400
4 °C / 12 weeks	12,800	200
RT / 1 week	12,800	1600
RT / 2–4 weeks	6400	800
RT / 5–6 weeks	3200	800
RT / 7 weeks	3200	400
RT / 8–10 weeks	1600	400
RT / 11 weeks	800	400
RT / 12 weeks	800	200
37 °C / 1 week	6400	800
37 °C / 2 weeks	1600	800
37 °C / 3 weeks	800	800
37 °C / 4 weeks	400	400
37 °C / 5–6 weeks	200	400
37 °C / 7 weeks	100	400
37 °C / 8 weeks	100	200
37 °C / 9 weeks	100	100
37 °C / 10–11 weeks	50	100
37 °C / 12 weeks	50	50

[a] Triplicate determinations

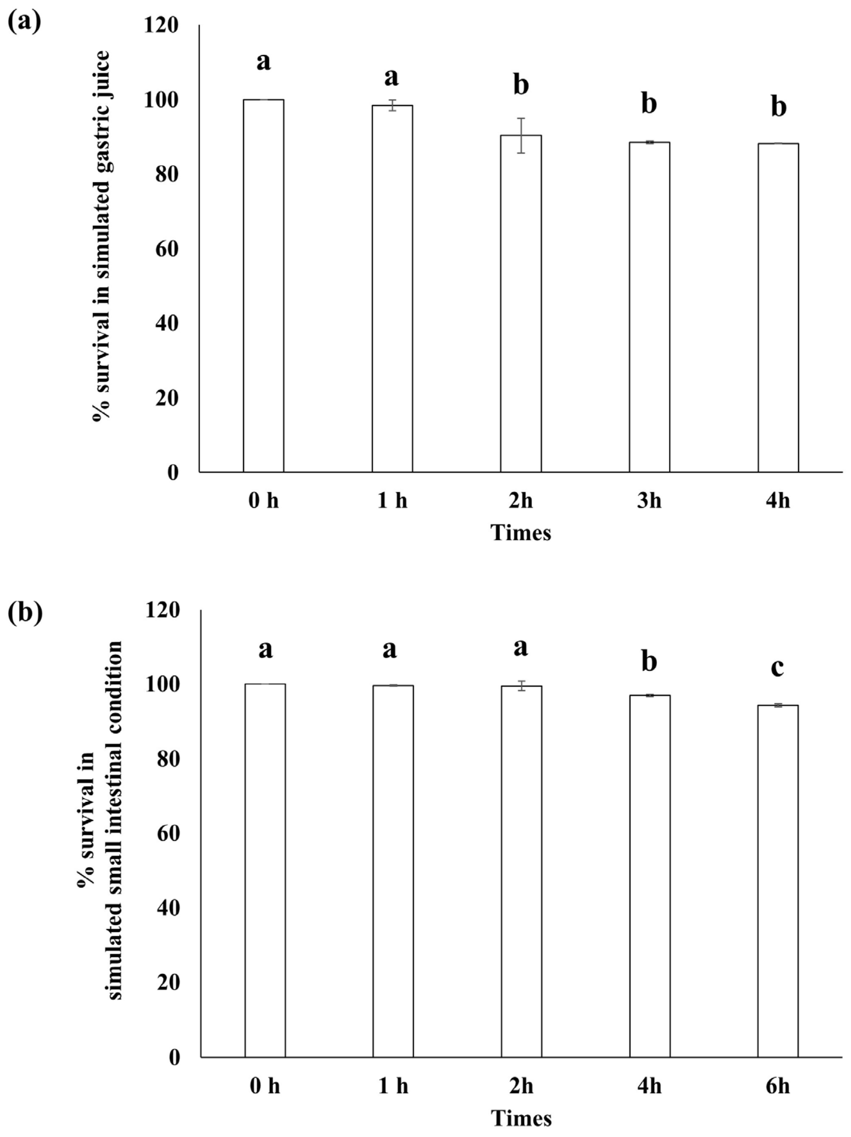

Figure S1. The percentage survival of *E. faecium* OV3-6 in simulated gastric juice (**a**) and simulated small intestinal condition (**b**). Data are shown as means ± SD of triplicate determinations. The different letters above columns indicate that the values are significantly different ($p < 0.05$).

50S ribosomal protein L29

MKVKEIR**ELTTAEMLDKEK**QLKEELFNLR**FQLATGQLENTAR**IKEVRQSIARIKTVLREQAN

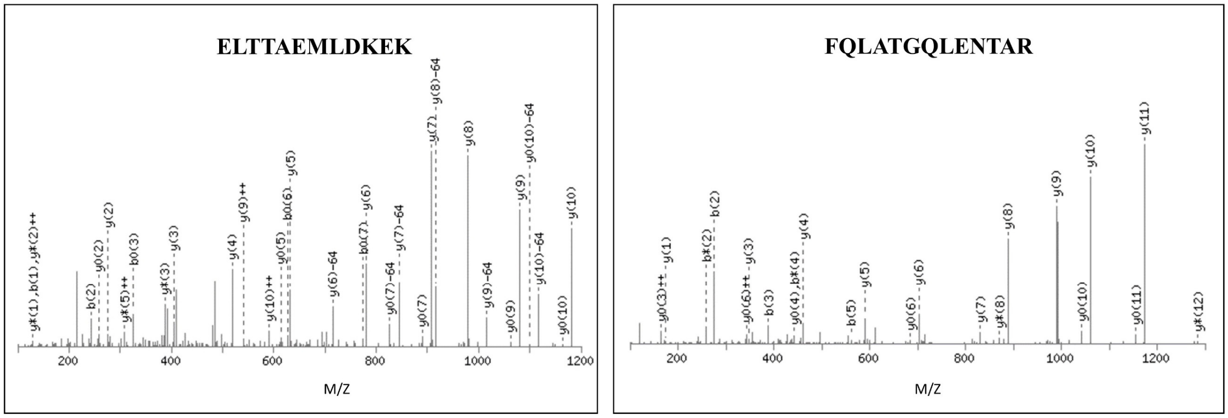

Figure S2. Mass spectrometry spectrum of amino acid sequence ELTTAEMLDKEK and F QLATGQLENTAR from ~10-16 kDa protein band of *E. faecium* OV3-6, which matches 50S ribosomal proteins L29 are shown in bold text.
